# Mobile Apps Designed for Patients With Polycystic Ovary Syndrome: Content Analysis Using the Mobile App Rating Scale

**DOI:** 10.2196/71118

**Published:** 2025-06-17

**Authors:** Zahra Arabkermani, Saeed Barzegari, Atefeh Rouhani, Nilofar Nahavandi, Omer Gibreel, Ibrahim Arpaci

**Affiliations:** 1 Department of Paramedicine, Amol School of Paramedical Sciences, Mazandaran University of Medical Sciences, Sari, Iran; 2 Student Research Committee, Mazandaran University of Medical Sciences, Farah Abad Street, 1454, Sari, 48175, Iran, 09369118477; 3 Management Information Systems, College of Business Administration, Gulf University for Science and Technology, Mishref, Kuwait; 4 Department of Software Engineering, Faculty of Engineering and Natural Sciences, Bandirma Onyedi Eylul University, Balıkesir, Turkey; 5 Department of Computer Science and Engineering, College of Informatics, Korea University, Seoul, Republic of Korea

**Keywords:** mobile apps, Mobile App Rating Scale, polycystic ovary syndrome, patient satisfaction, reproductive health

## Abstract

**Background:**

Digital health interventions, especially mobile apps, have become instrumental in helping women at risk of polycystic ovary syndrome (PCOS), increasing their understanding of the condition, improving self-care, and fostering empowerment. However, their rapid proliferation has brought about significant challenges regarding quality assessment and evidence-based determination. Therefore, establishing reliable quality assessment methods is essential to assist patients with PCOS in identifying effective and trustworthy mobile health tools.

**Objective:**

This study was designed to assess the content and quality of mobile apps developed for patients with PCOS using the Mobile App Rating Scale (MARS) to provide insights into their strengths, limitations, and areas needing improvement.

**Methods:**

In this descriptive-analytical study conducted in June 2024, a comprehensive search was performed to identify English and Persian mobile apps related to PCOS through the Café Bazaar and Google Play Store platforms, using both direct search methods and auxiliary tools such as AppAgg and AppBrain. Two trained reviewers (AR and NN) independently reviewed the apps using the MARS tool. The interrater reliability was measured using the intraclass correlation coefficient test. The quality of each app was scored across 4 dimensions: engagement, functionality, aesthetics, and information quality.

**Results:**

Of the initial 199 apps identified, 15 met the inclusion criteria after screening and updates. The interrater agreement rate was 85%, which is considered acceptable. The apps’ overall quality was sufficient, as assessed using the MARS, with a mean score of 3.6 (SD 0.52) of 5. Functionality and aesthetics emerged as the highest-scoring dimensions, highlighting user-friendliness and visual appeal (n=10). In contrast, engagement following information quality received the lowest average score, indicating limited interactivity and gaps in providing evidence-based information. The Ask PCOS app achieved the highest overall score, performing exceptionally well in subjective quality (4.75) and app-specific quality (4.33), reflecting its strong capacity to positively impact users’ knowledge, attitudes, and behaviors related to PCOS. Uvi Health and Ask PCOS scored highest in engagement (4.2), while PCOS & PCOD Diet & Remedies led in functionality (5), and Uvi Health topped aesthetics (5).

**Conclusions:**

The findings revealed that even though many available PCOS-related apps demonstrate strengths in technical performance and design, critical limitations persist regarding user engagement and the credibility of the information provided. The predominance of commercially affiliated apps without academic or clinical oversight was identified as a key contributing factor to these shortcomings. These results underscore the need for future app development to incorporate more user-engaging features, reliable evidence-based content, and personalization strategies to enhance user engagement and support effective PCOS self-management. Addressing these limitations and leveraging the capabilities of existing mobile devices are essential steps toward improving the overall quality and impact of mobile health interventions for individuals with PCOS.

## Introduction

Polycystic ovary syndrome (PCOS) is one of the most prevalent endocrine disorders among women of reproductive age, affecting 5% to 18% of this population [1]. It is associated with reproductive, metabolic, and psychological features, making it a significant public health concern [2]. Also known as hyperandrogenic anovulation [3], PCOS has been diagnosed in over 105 million women aged 15 to 49 years globally, with prevalence rates showing an upward trend over the past decade [4]. Additionally, the economic burden of this condition on society is substantial; annual health care costs related to PCOS are estimated to exceed US $4 billion in the United States alone [5].

The unknown etiology, diverse clinical presentations, complex pathophysiology, and challenges in accurately diagnosing PCOS have drawn considerable attention to this condition in various studies [6]. This chronic and heterogeneous disorder manifests with symptoms such as menstrual irregularities, infertility, hirsutism, acne, and obesity [7]. It is typically diagnosed when complications such as alopecia, acne, hair loss, and infertility begin to affect the patient’s quality of life significantly [8]. Restoration of gut microbiota through probiotics, prebiotics, or fecal microbiota transplant may be considered an innovative, effective, and noninvasive approach for preventing and alleviating the symptoms associated with PCOS [9]. In addition to these interventions, lifestyle modifications, effective stress management, weight reduction through an appropriate low-calorie diet, the use of combined oral contraceptives, medications such as metformin, and kisspeptin-based therapies may also play a crucial role in improving this condition [10,11]. However, the first step in managing PCOS is raising awareness and making an accurate diagnosis, as both contribute to an improved quality of life for patients [12]. Given the chronic nature of the disease, its negative impact on patients’ lives, and the need for lifelong treatment and monitoring, effective self-care is essential for managing PCOS and enhancing the quality of life [13]. According to international guidelines, lifestyle interventions (raising awareness, engaging in appropriate exercise, and following dietary plans) are also recommended as an initial treatment approach for managing the condition [14].

In this regard, digital health technologies, including mobile health (mHealth) apps, offer significant potential to enhance awareness and provide tailored interventions focused on self-care and empowerment for women at high risk of developing PCOS. Recent artificial intelligence (AI) advancements have enhanced health management tools for conditions like PCOS. AI systems, including machine learning algorithms and predictive models, enable early detection by analyzing symptoms, hormonal patterns, and biometric data. AI-powered apps offer personalized recommendations, predictive diagnostics, and chatbot support, empowering users to manage their health proactively. These technologies improve diagnostic accuracy and reduce delays in clinical interventions, ensuring timely and personalized care [15-17].

In recent years, the adoption of mHealth technologies for delivering health care services and managing chronic conditions, such as diabetes and PCOS, which necessitate various self-care measures following diagnosis, has experienced significant growth. Integrating educational resources and behavior-tracking features in mHealth apps may facilitate monitoring symptoms and reduce the likelihood of misreporting disease indicators. Moreover, maintaining a healthy lifestyle and adhering to a balanced diet are essential components of effective self-care for patients [15,18,19].

mHealth refers to mobile technology, such as smartphones, tablets, and wearable devices, to provide medical care, monitor health status, and educate patients, health care providers, and the public. According to studies, as of January 2021, approximately 4.66 billion internet users worldwide were actively using the internet, of which 92.6% (4.32 billion) accessed the internet through mobile devices such as smartphones and tablets [20]. This widespread access has contributed to the rapid expansion of mHealth technologies. Notably, mobile apps have shown significant advantages in raising awareness and supporting self-care among individuals with PCOS. Furthermore, there has been substantial progress in developing mHealth interventions to improve health outcomes and mitigate health risks associated with PCOS, highlighting the growing potential of these tools in managing chronic conditions [15].

Studies indicate that patients with PCOS who gain sufficient knowledge about their condition and have access to a secure platform for sharing personal experiences through mobile apps are likely to experience improvements in their quality of life, self-esteem, and confidence in managing their health [21,22]. An in-depth understanding of these apps’ self-care content is essential for informing strategies for enhancing such content [23]. Attention to the quality of mHealth apps is significant, as inaccurate or misleading information can significantly impact health-related decisions and outcomes. In addition to content, other quality attributes, including user-friendliness, satisfaction, functionality, and aesthetic appeal, should also be carefully considered [24]. As a result, further studies to evaluate both existing tools and those under development are essential [25].

One of the most widely used tools for evaluating mobile apps is the Mobile App Rating Scale (MARS), a reliable, validated, and easy-to-use tool for categorizing and assessing mHealth apps [26]. This tool consists of 23 items across objective and subjective sections, categorized into engagement, functionality, aesthetics, information quality, and subjective quality. Additionally, it includes 6 separate questions for app-specific quality evaluation. The MARS is widely recognized as a reliable and practical method for categorizing and evaluating the quality of mHealth apps [25]. Even though the number of mHealth apps has significantly increased, many have limited value and credibility, with only a tiny percentage being evidence-based or developed with professional medical involvement. Therefore, a growing need exists to evaluate their quality [23]. Users typically assess the quality of apps based on program descriptions, star ratings, and comments, which are unreliable criteria for measuring quality [27]. While various engaging and practical apps are available for PCOS, the lack of comprehensive evaluations of clinically oriented apps highlights the critical need for thorough research to assess the quality and efficacy of these apps. Such evaluations are essential for identifying useful and practical apps that provide adequate and relevant information regarding PCOS [28]. Accordingly, this study was designed to assess the characteristics and content of mobile apps developed for patients with PCOS using the MARS tool to provide a comprehensive overview of their current strengths and shortcomings and to inform future research and app development efforts.

## Methods

### Ethical Considerations

This descriptive-analytical study was conducted in June 2024 and received ethical approval from the ethics committee of Mazandaran University of Medical Sciences (approval code IR.MAZUMS.REC.1403.458). As the study did not involve direct interaction with human participants and focused solely on the evaluation of publicly available mobile apps, informed consent was not required. No personal or identifiable user data were collected during the study. All information analyzed was derived from publicly accessible sources such as app store descriptions and content. Therefore, issues related to privacy and confidentiality did not apply. Moreover, since no human participants were involved, no compensation was provided.

### Search Strategy and App Identification

In this descriptive-analytical study conducted in June 2024 after ethics approval (IR.MAZUMS.REC.1403.458), a systematic search was performed to identify all Persian- and English-language mobile apps related to PCOS to raise awareness or offer targeted interventions. Searches were initially conducted in Café Bazaar and Google Play Store to identify relevant apps, followed by using AppAgg and AppBrain tools for more precise retrieval of available apps in Google Play Store, as this platform provides different content depending on the region. Persian keywords and the English terms “PCOS,” “polycystic ovary syndrome,” and “polycystic ovarian syndrome” were used independently by 2 reviewers (AR and NN).

### Eligibility Criteria

The apps were selected using a purposive nonprobability sampling approach based on inclusion criteria. While this approach allowed for the focused selection of apps aligned with our inclusion criteria, it may limit generalizability and introduce selection bias, as not all potentially relevant apps could be captured. The inclusion criteria for apps chosen in this study were as follows: (1) availability on Café Bazaar and the Google Play Store, (2) use of either Persian or English language, (3) a specific focus on PCOS, (4) being free of charge, (5) compatibility with the Android operating system (due to iOS limitations in Iran), and (6) provision of interventions aimed at enhancing awareness or improving quality of life. Conversely, the exclusion criteria encompassed apps that did not specifically address PCOS, were compatible solely with the iOS operating system, required in-app purchases immediately upon entry and before accessing features, or were restricted to outdated, noninstallable versions.

### Data Extraction and Analysis

After applying the inclusion and exclusion criteria, irrelevant and duplicate apps were removed. Because the focus was not on PCOS, all Persian-language and several English-language apps that did not meet the specified standards were excluded. Two trained reviewers (AR and NN) identified and installed 18 apps designed explicitly for PCOS on smartphones.

Following their training on the MARS, each reviewer thoroughly examined the app’s characteristics and features after installation. This process involved reading the description of apps provided by the developers in app markets, creating an account if required, and inputting test data to check the app’s functionality. The evaluation was initially conducted independently by reviewers (AR and NN), with a third reviewer (ZA) involved in case of any disagreements to maintain consistency. The assessments from both reviewers (AR and NN) were used to examine the level of agreement in the MARS assessment.

Based on the consensus reached between the reviewers (AR and NN), the data and evaluations provided by the reviewers (AR and NN) were subsequently used to generate the remaining review results. The data were organized in a Microsoft Excel spreadsheet. Descriptive data and frequency analyses were conducted to evaluate app features, including the intended target audience, the strategies provided, Google Play Store metrics such as ratings (on a 5-star scale) and the number of user reviews, as well as the involvement of commercial, academic, or professional contributors in the development of the app and the nature of interventions for managing the condition. The overall quality of each app was assessed by calculating the average scores from the MARS subscales (as outlined in the Mobile App Rating Scale section). In the months before completing the review in late September, the apps included in the study were re-evaluated to update the data and confirm the accuracy of the entries.

### Mobile App Rating Scale

MARS is a straightforward and reliable tool for classifying and evaluating the quality of mobile apps. It consists of four sections: (1) app classification, (2) app quality assessment, (3) app subjective quality, and (4) app-specific evaluation. The validity and reliability of MARS have been confirmed in a study by Terhorst et al [26], titled “Validation of the Mobile Application Rating Scale.” Additionally, this tool has been culturally adapted in Iran [29].

The app classification section gathers essential descriptive and technical information about the app, such as its focus area (eg, depression and physical health), theoretical background (eg, cognitive behavioral therapy, behavioral methods, and relaxation), affiliation (eg, commercial and governmental), target age range, and technical features (eg, sharing options and inclusion of an app-based community). The app quality section comprises 19 questions that assess quality across four dimensions as follows:

1. Engagement: Five questions regarding evaluating the app’s ability to captivate users through features that are interesting, customizable, and engagement-driven, such as alerts, messages, reminders, feedback, and sharing options tailored for the target audience.
2. Functionality: Four items assessing ease of use, navigability, logical flow, and gestural design.
3. Aesthetics: Three items about reviewing graphic design, overall visual appeal, color scheme, and consistency of style.
4. Information quality: There are 7 items regarding evaluating the inclusion of high-quality information, ensuring it originates from credible sources.

Meanwhile, the subjective quality section includes 4 questions focused on user satisfaction, overall impression, and likelihood to recommend the app to those who might benefit. The app-specific section consists of 6 items that measure the app’s influence on users’ awareness, knowledge, attitudes, intentions to change, help-seeking behaviors, and behavior-related changes related to the topic being addressed. Each item was scored on a scale from 1=insufficient to 5=excellent. All apps were assessed using the MARS, and each section’s average scores were calculated. The overall quality of the apps was derived from the mean scores of the engagement, functionality, aesthetics, and information quality dimensions. The average score for the subjective quality section was considered independently from the overall mean score, enhancing the tool’s capacity for evaluating objective quality. Like the subjective quality section, the app-specific section was considered separately on a scale of 1‐5 to assess the intended health behavior change it aims to promote. Data analysis was conducted using STATA (version 17; StataCorp LLC) software.

### Reliability and Agreement

To assess reliability, reviewers (AR and NN) on the Google Play Store independently searched for apps related to PCOS. Two trained reviewers (AR and NN) initially installed and evaluated 5 apps, spending 30 minutes on each to establish consensus. The results were then discussed and analyzed collaboratively. If either reviewer faced issues accessing or downloading an app, a third reviewer (ZA) was consulted to determine if the app should be included and to conduct the review if required. To ensure consistency in evaluations, the intraclass correlation coefficient was used to evaluate the agreement between the 2 reviewers (AR and NN; *r*≥0.81‐1.0=excellent, 0.61‐0.80=particularly good, 0.41‐0.60=good, 0.21‐0.40=moderate, and 0.2‐0.0=poor) [30,31].

## Results

### Overview

Following the search process, 199 were identified in app markets. After removing the duplicates (n=8), additional screening, and applying inclusion and exclusion criteria, 18 apps were downloaded for evaluation. During the second round of examination of the apps, which involved checking for updates to the latest versions, it was found that 3 apps were no longer available on Google Play. These apps thus had to be excluded; hence, the final sample of analysis apps remained 15 in number.

### App Characteristics

Among the 15 apps analyzed, the majority (n=11, 73%) were classified under the health and fitness category on the Google Play Store. At the same time, 3 (20%) apps fell under the food and drink category, and 1 (7%) was listed under the education category. In terms of affiliations, 12 (80%) apps were commercially affiliated, 1 (7%) was linked to an academic institution, and 2 (13%) had unknown affiliations. All apps offered a free version; however, 11 (73%) included in-app purchases to access additional features. A total of 1 app required a premium subscription for polycystic ovarian syndrome to access almost 90% of its features, while 4 (27%) apps were entirely free. The number of downloads varied considerably. In total, 13 (87%) apps had been downloaded more than 1000 times, while 2 (13%) apps had fewer downloads: Ovara with over 50 downloads and Revaiv with over 500 downloads.

Among the apps that received a rating of above 4 stars (n=5, 33%), Uvi Health: PCOS Diet & Fitness (4.4) and ASK PCOS (5) ranked the highest; in contrast, 4 (27%) apps received ratings below 2 stars, of which Insulin Resistance Diet for PCOS obtained the lowest rating with 1 star. Remarkably, 33% (n=5) had over 100 user reviews, and about equal proportions had fewer than 10. Over 60% (n=10) of the apps had been updated to their most recent versions in the last year, reflecting active developer engagement. Tables1  2 provide a detailed summary of these characteristics.

**Table 1.** General characteristics of the apps: availability, ratings, and user engagement metrics.

	App name	Version	Last update	Downloads	Star	Number of ratings
1	Insulin Resistance Diet for PCOS	1.0.1	October 16, 2018	>10,000	1	34
2	MIRA Health	1.8.7	June 5, 2024	>1000	N/A^a^	N/A
3	PCOS Tracker	3.1	June 3, 2024	>10,000	2.5	105
4	Uvi Health: PCOS Diet & Fitness	1.1.0	June 11, 2024	>50,000	4.4	195
5	Revaiv	1.0.2406250	June 4, 2024	>500	N/A	N/A
6	PCOS & PCOD Diet Plan Recipes	2.1.1‐45	April 24, 2024	>10,000	3.7	87
7	PCOS Mantra	2.2.1	February 7, 2024	>5000	4.1	9
8	PCOS & PCOD Diet & Remedies	1.7	April 1, 2024	>10,000	3.7	130
9	Ask PCOS	2.0.0.0	December 5, 2021	>5000	5	4
10	Yoga for PCOS-AI Exercise	9.5	April 5, 2024	>50,000	4.3	650
11	Easy PCOS Diet Cookbook	1.3.1.3	October 23, 2023	>5000	2.3	6
12	Polycystic Ovarian Syndrome	1.0.2	October 4, 2021	>5000	2.9	9
13	Veera Health	1.12.53	June 10, 2024	>10,000	3.9	169
14	PCOS	6.0	May 13, 2024	>1000	4	7
15	Ovara	1.5.0	October 12, 2023	>50	N/A	N/A

a N/A: not applicable.

**Table 2.** General characteristics of the apps: affiliation and accessibility features.

	App name	Affiliation	Initial cost	In-app purchase	Category in the store
1	Insulin Resistance Diet for PCOS	Commercial	Free	Yes	Food and Drink
2	MIRA Health	Commercial	Free	Yes	Health and Fitness
3	PCOS Tracker	Commercial	Free	No	Health and Fitness
4	Uvi Health: PCOS Diet & Fitness	Commercial	Free	Yes	Health and Fitness
5	Revaiv	Commercial	Free	Yes	Health and Fitness
6	PCOS & PCOD Diet Plan Recipes	Commercial	Free	Yes	Food and Drink
7	PCOS Mantra	Commercial	Free	No	Health and Fitness
8	PCOS & PCOD Diet & Remedies	Unknown	Free	Yes	Health and Fitness
9	Ask PCOS	Academic	Free	No	Health and Fitness
10	Yoga for PCOS-AI Exercise	Commercial	Free	Yes	Health and Fitness
11	Easy PCOS Diet Cookbook	Unknown	Free	Yes	Food and Drink
12	Polycystic Ovarian Syndrome	Commercial	Free	Yes	Education
13	Veera Health	Commercial	Free	Yes	Health and Fitness
14	PCOS	Commercial	Free	Yes	Health and Fitness
15	Ovara	Commercial	Free	No	Health and Fitness

### App Classification Based on MARS

The reviewed apps primarily focused on physical health (15/15, 100%), with over half (8/15, 53%) also targeting behavior change. Among these, only 1 (7%) app had PCOS & PCOD Diet Plan Recipes aimed at mindfulness and relaxation, with 2 focusing on reducing negative emotions. The apps used diverse strategies to reach their objectives, with the most common approach being education and behavioral skill development. Specifically, 80% (12/15) of the apps used educational content, with an identical share providing tips, advice, or strategies to help users build behavioral skills.

Most apps (n=12) included educational content and interventions, such as providing recipes, exercise routines, or consultations to help manage the condition. However, 2 apps, Polycystic Ovarian Syndrome and PCOS & PCOD Diet & Remedies, focused exclusively on raising awareness without offering any interventions. On the other hand, 20% (3/15) of the apps were dedicated solely to interventions aimed at managing or mitigating the symptoms of PCOS. Among the apps offering interventions, 27% (4/15) provided premium options for accessing additional features related to symptom management.

Regarding support features, 67% (10/15) of the apps offered symptom or activity-tracking tools to help users monitor their health. Half of the apps (7/15, 47%) included community engagement features, allowing users to share experiences or connect with others. Additionally, 33% (5/15) incorporated mindfulness or relaxation techniques into their interventions.

A significant proportion of the apps (11/15, 73%) required internet access. Sharing capabilities, which allow users to engage with communities or share health data, were available in 60% (9/15) of the apps. Moreover, 60% (9/15) of the apps implemented password protection or login requirements to enhance user security. Notification systems (reminders) were present in 40% (6/15), aiding users in maintaining adherence to health goals through reminders—all apps catered to a general audience, with 1 specifically targeting users aged 17 years and older. None explicitly focused on adolescents or older people. Details are presented in Tables3  4.

**Table 3.** Focus areas and technical features identified in the app classification section of Mobile App Rating Scale.

	App name	Focus or objective	Target age group	Technical aspect	Internet access
1	Insulin Resistance Diet for PCOS	Physical health	Everyone	Allows sharing	No
2	MIRA Health	Behavior change, physical health, other	Everyone	Allows sharing and password protection and requires a login	Yes
3	PCOS Tracker	Physical health, other	Everyone	Allows sharing and sends notifications	Yes
4	Uvi Health: PCOS Diet & Fitness	Behavior change, physical health, other	Everyone	Allows sharing, requires login, and has a community	Yes
5	Revaiv	Physical health	Everyone	Requires login and sends a reminder	Yes
6	PCOS & PCOD Diet Plan Recipes	Mindfulness or meditation, relaxation, behavior change, physical health	Everyone	Allows sharing, sends reminders, password protection, and requires a login	No
7	PCOS Mantra	Physical health, other	Everyone	Password protection and login are required	Yes
8	PCOS & PCOD Diet & Remedies	Physical health, other	Mature 17+	No technical feature	No
9	Ask PCOS	Reducing negative emotions, behavior change, goal setting, physical health	Everyone	Allows sharing and password protection, requires login, and has a community	Yes
10	Yoga for PCOS-AI Exercise	Behavior change, physical health	Everyone	Sends reminders and requires login	Yes
11	Easy PCOS Diet Cookbook	Behavior change, physical health	Everyone	No technical aspect	No
12	Polycystic Ovarian Syndrome	Physical health	Everyone	No technical aspect	Yes
13	Veera Health	Physical health, other	Everyone	Allows sharing, sends a reminder, requires a login, and has a community	Yes
14	PCOS	Reducing negative emotions, behavior change, physical health, other	Everyone	Allows sharing, sends reminders, password protection, requires login, and has a premium community option	Yes
15	Ovara	Behavior change, physical health, other	Everyone	Allows sharing and password protection and requires a login	Yes

**Table 4.** Strategies and interventions of the analyzed apps from the Mobile App Rating Scale app classification section.

	App name	Strategies or theoretical background	Intended intervention
1	Insulin Resistance Diet for PCOS	Information or education, advice or tips or strategies or skill training	Enhances awareness and provides recipes or diet
2	MIRA Health	Information or education, monitoring or tracking, advice or tips or strategies or skills training	Enhances awareness, provides recipes or exercises, and tracks daily progress and activity
3	PCOS Tracker	Information or education, monitoring or tracking	Tracks the symptoms and raises awareness via linking to articles and videos
4	Uvi Health: PCOS Diet & Fitness	Assessment, information or education, monitoring or tracking, goal setting, advice or tips or strategies or skills training, mindfulness or meditation	Tracks cycles, raises awareness, provides recipes and exercises, and consults with doctors
5	Revaiv	Monitoring or tracking	Tracks cycle and daily progress, activity, and mood and offers premium plans, including recipes and exercises
6	PCOS & PCOD Diet Plan Recipes	Information or education, monitoring or tracking, advice or tips or strategies or skills training, mindfulness or meditation	Offers recipes and basic photo-based yoga, raises awareness, and tracks activities
7	PCOS Mantra	Information or education, monitoring or tracking, advice or tips or strategies or skills training, mindfulness or meditation, relaxation	Tracks symptoms, raises awareness, and offers exercise and massage-based consultation
8	PCOS & PCOD Diet & Remedies	Information or education, advice or tips or strategies or skills training, relaxation	Enhances awareness
9	Ask PCOS	Information or education, monitoring or tracking, advice or tips or strategies or skills training, CBT^a^, strength-based	Enhances awareness, tracks symptoms, activities, and cycle, and provides a forum for sharing experiences and a self-assessment quiz of the user’s status
10	Yoga for PCOS-AI Exercise	Monitoring or tracking, advice or tips or strategies or skills training, goal setting, mindfulness or meditation, relaxation	Provides exercises and yoga plans for different levels, with some of them being premium, tracks activities, and offers basic diet plans
11	Easy PCOS Diet Cookbook	Information or education, advice or tips or strategies or skills training	Raises awareness and provides recipes
12	Polycystic Ovarian Syndrome	Information or education	Enhances basic awareness of the condition
13	Veera Health	Assessment, feedback, advice or tips or strategies or skills training, goal setting, monitoring or tracking, premium feedback strategy	Tracks cycle and progress process and provides premium consultation, diet, and fitness plan
14	PCOS	Information or education, feedback, advice or tips or strategies or skills training, goal setting	Enhances awareness, provides recipes, takes a quiz to offer supplements that best suit the user’s needs, has a low-engagement discussion board, and offers message-based consultation
15	Ovara	Information or education, strength-based	Enhances awareness, provides exercise videos and recipes, and shares stories of others dealing with this condition

a CBT: cognitive behavioral therapy.

### MARS Score

#### Overview

All apps were systematically reviewed and evaluated using the MARS tool, achieving an interrater agreement of 85%, reflecting high consistency between reviewers (AR and NN). Two trained reviewers (AR and NN) initially rated 5 apps collaboratively to align their interpretation of the MARS criteria. The remaining apps were assessed independently. When scores differed, the reviewers (AR and NN) discussed their assessments to reach a consensus, with a third reviewer (ZA) consulted if necessary. In agreement cases, the shared score was used in the final analysis. As shown in Table 5, the mean score of quality, derived from the average of the 4 core MARS domains (engagement, functionality, aesthetics, and information quality), was 3.68 (SD 0.56), indicating that the overall quality of the apps ranged from moderate to high. Evaluating the included apps using the MARS provided insights into their strengths and weaknesses across key domains.

**Table 5.** Mobile App Rating Scale scores of the evaluated apps.

App name	Engagement	Functionality	Aesthetics	Information quality	Mean score (SD)	Subjective quality	App-specific
Insulin ‎Resistance Diet ‎for PCOS	2.20	4.50	3.00	3.60	3.32 (0.81)	2.25	3.00
MIRA Health	4.20	4.50	4.66	3.20	4.14 (0.62)	3.50	4.00
PCOS Tracker	3.60	4.50	3.33	3.80	3.80 (0.76)	2.25	2.50
Uvi Health: PCOS Diet & Fitness	4.20	4.50	5.00	4.00	4.42 (0.44)	4.50	4.00
Revaiv	3.20	4.00	4.00	2.20	3.35 (0.76)	2.25	2.33
PCOS & PCOD Diet Plan Recipes	4.20	4.75	3.66	3.40	4.00 (0.52)	4.00	3.33
PCOS Mantra	3.00	3.50	3.33	3.00	3.20 (0.44)	2.25	2.83
PCOS & PCOD Diet & Remedies	2.00	5.00	2.66	2.80	3.11 (1.06)	2.25	3.00
Ask PCOS	4.20	4.50	4.66	4.57	4.48 (0.26)	4.75	4.33
Yoga for PCOS-AI Exercise	3.80	4.25	4.00	2.20	3.56 (0.84)	2.50	2.66
Easy PCOS Diet Cookbook	2.40	4.50	3.33	3.40	3.40 (0.69)	3.00	3.33
Polycystic Ovarian Syndrome	1.80	4.00	2.00	3.00	2.63 (1.08)	1.25	1.83
Veera Health	3.80	3.75	4.33	3.75	3.90 (0.55)	2.50	3.00
PCOS	3.80	4.75	4.66	4.16	4.35 (0.48)	3.75	3.83
Ovara	3.00	4.25	3.66	3.50	3.60 (0.49)	3.25	3.33
Total, mean (SD)	3.29 (0.87)	4.35 (0.42)	3.75 (0.83)	3.37 (0.72)	3.68 (0.56)	2.95 (1.01)	3.15 (0.65)

#### MARS Quality Score

Functionality was the highest-performing category, with an average score of 4.35. Two apps, PCOS & PCOD Diet Plan Recipes and PCOS, achieved the maximum score of 4.75. In contrast, PCOS Mantra received the lowest functionality score of 3.50, which still indicates an acceptable level of functionality. Remarkably, over 80% (n=13) of the apps scored 4.00 or higher in this domain, highlighting high functionality and ease of use among the apps.

Following that, aesthetics, which evaluates the visual and structural design of the apps, scored an average of 3.75. Uvi Health: PCOS Diet & Fitness reached the maximum score of 5.00, demonstrating excellence in appeal and overall design regarding the user interface. In contrast, Polycystic Ovarian Syndrome had the lowest rating at 2.00, indicating significant visual and design appeal shortcomings. In total, 87% (n=13) of the apps achieved a score of 3.00 or higher, reflecting overall generally favorable design among the apps.

Engagement received the lowest mean score of 3.29 (SD 0.87). Almost one-quarter of the apps received the highest score of 4.20 in this domain. In contrast*,* Polycystic Ovarian Syndrome received the lowest score of 1.80, showing significant limitations in user engagement features. In total, 11 apps scored above 3.00, indicating that most apps provided satisfactory engagement. Information quality, with a slightly higher score of 3.37 compared to engagement, was the second-lowest domain, suggesting that while most apps offered helpful information, there were significant gaps in terms of quality and accuracy. Ask PCOS achieved the highest score in this domain, with 4.57, offering reliable and evidence-based content. In contrast, Revaiv and Yoga for PCOS-AI Exercise received the lowest scores of 2.20.

Overall, Ask PCOS received the highest overall quality score with 4.48, reflecting strong performance across most domains. Uvi Health: PCOS Diet & Fitness followed closely with a mean score of 4.42 (SD 0.44). On the other hand, Polycystic Ovarian Syndrome had the lowest mean score of 2.63 (SD 1.08), indicating poor performance in engagement, aesthetics, and information quality. Overall, except for the Polycystic Ovarian Syndrome app, which had the lowest mean score of 2.63 (SD 1.08), the remaining apps achieved a mean score above 3.00, indicating that they were acceptable to good in quality.

#### Subjective Quality and App-Specific Quality

The subjective quality domain had an average score of 2.95, while the app-specific features domain averaged 3.15. Ask PCOS excelled in both, scoring the highest in subjective quality with 4.75 and app-specific features with 4.33, reflecting its intense user satisfaction and effectiveness in promoting positive behavioral change for PCOS management. On the other hand, Polycystic Ovarian Syndrome scored the lowest in both domains, with 1.25 in the former and 1.83 in the latter, highlighting poor user satisfaction and the nonavailability of specific features to encourage improvements in disease management practices. In total, 50% (n=7) of the apps scored above 3.00 in subjective quality, and 10 (67%) apps scored above 3.00 in app-specific features, indicating moderate satisfaction. MARS scoring for PCOS programs is shown in Table 5.

## Discussion

### Principal Findings

Although the number of appealing and practical mHealth apps for PCOS has significantly increased, little is known about their quality, content, focus, comprehensiveness, and potential to drive behavioral change. To address this gap, this study conducted the first comprehensive and systematic evaluation of PCOS-related mHealth apps available on the Google Play Store. The findings indicate that while most apps demonstrated strong functionality and appealing aesthetics, essential gaps remained in user engagement and information quality. Although the majority were technically sound and easy to use, many lacked user-engaging features and credible, evidence-based content essential for effective self-management. Overall, most apps exhibited a moderate level of quality, with only a few demonstrating excellence.

### MARS Evaluation

As highlighted in the results, the 15 evaluated mobile apps demonstrated notable strengths in functionality (4.35) and aesthetics (3.75), which received the highest scores among the MARS quality domains. These findings underscore developers’ emphasis on creating user-friendly and visually appealing interfaces, crucial for enhancing the initial user experience and facilitating sustained use. Thus, generally, apps were user-friendly, navigable, and well-designed. These findings align with previous studies, highlighting functionality and aesthetics as key strengths of health-focused mobile apps [23,32-36].

However, the engagement domain revealed the lowest average score, a critical limitation. Unlike other studies where information quality often received the weakest ratings [24,35-38], the lower engagement scores in this evaluation highlight specific deficiencies, such as limited personalization, minimal dynamic features, and insufficient feedback mechanisms, which restrict opportunities for meaningful user interaction, a factor vital for maintaining long-term interest and adherence. Engagement deficiencies resonate with similar findings from evaluations of health apps for mental health and menstrual pain management [20,23,25,27,39], where the lack of interactivity and tailored content was a common challenge. Addressing these gaps by incorporating gamification, dynamic feedback, and adaptive content could significantly improve user satisfaction and retention.

While performing slightly better than engagement, the information quality subscale exposed significant limitations in including credible, evidence-based content. Due to their commercial origins, many apps lacked integration with scientific resources or expert opinions, which are essential for providing reliable information. To address this, collaboration with academic institutions and adherence to standardized guidelines are recommended.

Overall, while the evaluated apps demonstrate high functionality and aesthetic appeal, they lack sufficient engagement features and evidence-based information. This dichotomy highlights the need for a balanced development approach integrating technical excellence with credible content and user-centric design. These deficiencies could thus be improved for more sustained behavior change and improved outcomes for people managing PCOS.

Finally, subjective and app-specific evaluations indicated limited user approval and moderate potential for behavior change. The subjective quality revealed a few apps demonstrating the potential for frequent use or recommendation. This reflects a gap between developer intentions and user expectations, emphasizing the need for better alignment with user needs. With average scores of 3.15 in the app-specific domain, many apps failed to motivate users or offer actionable strategies. These findings underscore the importance of integrating credible content, tailored feedback, and user-centric design to balance technical excellence with practical utility. These findings align with similar evaluations in previous studies [23,25,33,36] on menstrual symptom apps, where apps with personalized, user-engaging features with tailored feedback and evidence-based content achieved higher scores, while those lacking user-centric designs underperformed.

### App Characteristics Evaluation

Evaluating health apps requires ensuring they are evidence-based and supported by reliable information to guarantee safety, effectiveness, and informed decision-making [40-42]. A key finding of this study is that 12 of 15 apps were commercially affiliated, a trend that raises concerns about the lack of evidence-based content. Commercially driven apps often prioritize marketability over scientific accuracy, which can compromise the quality of information provided to users. This aligns with the previous study on asthma apps, which found that commercial apps are more likely to fail in integrating credible, evidence-based resources [33]. Only 1 app in this study had an academic affiliation, further highlighting the lack of collaboration with health care professionals, which is essential for ensuring the reliability of health apps.

The absence of scientific support in these apps can be attributed to the fast-paced development cycles of commercial apps, which often outpace the slower, more deliberate processes of academic research and validation [25]. While commercial apps benefit from rapid market entry, they frequently lack scientific validation to ensure their effectiveness for health-related conditions. As suggested by previous studies, stronger collaboration between developers and academic institutions or health care professionals is critical to improving health apps’ credibility, accuracy, and reliability [33,35,36].

Lifestyle modification is the primary treatment for overweight and obese women diagnosed with PCOS. The development of mobile apps aimed at enhancing motivation, providing lifestyle counseling, and offering evidence-based information can significantly benefit women affected by PCOS [43]. The apps reviewed in this study had a range of objectives. The most common objective identified among the reviewed apps was improving physical health (n=15) and behavior modification (n=8), while only 3 focused on mindfulness and reducing negative emotions. This emphasis on physical health aligns with broader trends in app development for pain and chronic condition management, where physical health goals often take precedence [25,36]. These objectives, while varied, can be beneficial for symptom management and treatment support when combined with lifestyle modification and implemented under medical supervision. This effectiveness, however, is achieved when used under the guidance of a health care professional.

All reviewed apps except 1 were designed for general audiences, with no focus on specific age groups, such as adolescents or older adults. This general approach may reduce the relevance of these apps for diverse user needs, particularly younger users who may require more digital and educational features. This broad focus follows trends observed in apps for chronic diseases, such as asthma, or those targeting mental health, where developers often cater to general populations without addressing the nuanced needs of diverse demographics [27,33].

The comparison between MARS scores and user-provided ratings and reviews revealed inconsistencies. Apps with high MARS scores often lacked substantial user ratings (Ask PCOS), while those with more reviews or higher star ratings occasionally scored poorly in technical evaluations (Uvi Health) [23,33]. In contrast, a few apps (Polycystic Ovarian Syndrome) with lower MARS scores had similarly low star ratings. This finding aligns with the study by Adrian Escriche-Escuder et al [25] on low back pain–related apps, highlighting that MARS ratings rely on technical criteria, whereas user ratings are often subjective [25]. Therefore, MARS scores and user ratings should be considered to better understand app quality.

This discrepancy highlights the difference between perceived quality (user ratings) and a more objective technical assessment of app quality provided by the MARS score. Users may prioritize high star ratings based on ease of use or personal experience, even if they lack solid technical foundations, potentially overlooking apps with better evaluations but fewer reviews. Therefore, MARS scores and user ratings should be considered together for a more comprehensive understanding of app quality. Relying on just 1 may lead to misguided conclusions about an app’s effectiveness.

While 60% (9/15) of apps were updated within the past year, Ask PCOS scored the highest despite not being updated since 2021, showing that strong initial design and evidence-based content can still lead to high quality. Strong foundational content and design can help an app maintain its high value despite a lack of recent updates. However, regularly updated apps tended to score better in functionality and aesthetics, aligning with some findings that updates improve usability and user satisfaction. These updates are often crucial for enhancing usability, addressing bugs, and incorporating user feedback, which ensures long-term user engagement.

Apps with outdated versions often scored lower, particularly in functionality, emphasizing the importance of ongoing developer engagement [44,45]. This highlights a broader issue affecting user trust, as they could be considered unreliable. As technology evolves, frequent updates ensure that apps remain relevant and compatible with new operating systems and devices, which is crucial for maintaining user engagement and retention.

Developers should focus on evidence-based content for a high-quality PCOS app, ensuring collaboration with health care professionals for reliable information. Engaging features like personalized tracking, adaptive content, and feedback mechanisms will enhance user involvement and retention. Regular updates are crucial to maintaining functionality and compatibility with evolving technologies. A well-designed, intuitive interface promotes ease of use, while strong privacy and security measures are essential for user trust. Integrating tools like goal setting, reminders, community support, and wearable device compatibility will further enhance the app’s effectiveness in managing PCOS.

### Limitations

The primary limitation of this study was the exclusion of paid and iOS-based apps. Due to geopolitical constraints and infrastructural limitations, consistent access to the Apple App Store was not feasible. Accordingly, the analysis was restricted to Android apps available through Google Play and Café Bazaar, the dominant platforms used within the study region. While this ensured contextual relevance and accessibility, it may have limited the scope of the analysis and resulted in missing relevant apps. Moreover, app marketplaces are highly dynamic, unpredictable, and frequently changing, so new and better apps may have emerged after the research was conducted.

Many of the free apps in this study were commercially affiliated, which raises concerns about potential biases in the information provided. This issue could be more pronounced in paid apps, which were not included.

Future research should expand the scope to include iOS and paid apps and use longitudinal designs to assess real-world effectiveness and user engagement. Furthermore, integrating user feedback with standardized technical assessments, such as the MARS, would enable a more comprehensive evaluation of quality, usability, and satisfaction.

### Conclusions

This study conducted a thorough analysis and content review of mobile apps related to PCOS to assist patients and health care professionals in identifying and recommending the highest-quality and most effective tools. The findings revealed that the reviewed apps were of average overall quality, highlighting a need for user interaction and informational content improvement to enhance their impact and reliability. While functionality and aesthetics were rated higher, engagement and information quality remained key areas of concern.

Ask PCOS achieved the highest overall quality and mean scores in subjective quality and app-specific features. Other well-rated apps, such as PCOS, Uvi Health, MIRA Health, and PCOS & PCOD Diet Plan Recipes, performed above average on the MARS, particularly in functionality and aesthetics. Notably, over 80% (n=12) of the apps scored highly in functionality, reflecting appropriate design and ease of use, which are critical for compelling user experiences.

Despite the vast number of PCOS-related apps available on the market, this study revealed that many scored low in informational quality and engagement, limiting their effectiveness for individuals with PCOS. These findings emphasize the need for significant improvements and updates to support broader and more impactful user adoption. Enhancing interactivity through personalized features, feedback mechanisms, and engaging content could improve user satisfaction and promote long-term adherence to these tools.

## Abbreviations

AI: artificial intelligence
MARS: Mobile App Rating Scale
mHealth: mobile health
PCOS: polycystic ovary syndrome
